# Relational interventions in psychotherapy: development of a therapy process rating scale

**DOI:** 10.1186/s12888-016-1021-4

**Published:** 2016-09-06

**Authors:** Randi Ulberg, Elisabeth Ness, Hanne-Sofie Johnsen Dahl, Per Andreas Høglend, Kenneth Critchfield, Phelix Blayvas, Svein Amlo

**Affiliations:** 1 Insititute of Clinical Medicine, Division of Mental Health and Addiction, University of Oslo, Box 85 Vinderen, 0319 Oslo, Norway; 2 Research Unit, Division of Mental Health and Addiction, Vestfold Hospital Trust, PO Box 2169, 3125 Tønsberg, Norway; 3 James Madison University, Harrisonburg, Virginia USA; 4 Department of Psychiatry, Vestre Viken Hospital Trust, Drammen, Norway

**Keywords:** Relational interventions, Transference, Psychodynamic, In-session process

## Abstract

**Background:**

In psychodynamic psychotherapy, one of the therapists’ techniques is to intervene on and encourage exploration of the patients’ relationships with other people. The impact of these interventions and the response from the patient are probably dependent on certain characteristics of the context in which the interventions are given and the interventions themselves. To identify and analyze in-session effects of therapists’ techniques, process scales are used. The aim of the present study was to develop a simple, not resource consuming rating tool for in-session process to be used when therapists’ interventions focus on the patients’ relationships outside therapy.

**Methods:**

The present study describes the development and use of a therapy process rating scale, the Relational Work Scale (RWS). The scale was constructed to identify, categorize and explore therapist interventions that focus on the patient’s relationships to family, friends, and colleges Relational Interventions and explore the impact on the in-session process. RWS was developed with sub scales rating timing, content, and valence of the relational interventions, as well as response from the patient. For the inter-rater reliability analyzes, transcribed segments (10 min) from 20 different patients were scored with RWS by two independent raters. Two clinical vignettes of relational work are included in the paper as examples of how to rate transcripts from therapy sessions with RWS.

**Results:**

The inter-rater agreement on the RWS items was good to excellent.

**Conclusion:**

Relational Work Scale might be a potentially useful tool to identify relational interventions as well as explore the interaction of timing, category, and valence of relational work in psychotherapies. The therapist’s interventions on the patient’s relationships with people outside therapy and the following patient-therapist interaction might be explored.

**Trial registration:**

First Experimental Study of Transference-interpretations (FEST307/95)

Registration number: ClinicalTrials.gov Identifier: NCT00423462.

**Electronic supplementary material:**

The online version of this article (doi:10.1186/s12888-016-1021-4) contains supplementary material, which is available to authorized users.

## Background

There is a growing emphasis on the development of personalized treatment for mental disorders [1]. Several studies have shown effect of different psychotherapies for the most frequent disorders as anxiety and depression. Two of the mostly used and researched therapy modes for these disorders are Cognitive Behavior Therapy (CBT) and Psychodynamic Therapy (PDT). Both CBT and PDT are repetitively shown to be more effective than no treatment. Different psychotherapy modalities work, on average, equally well [2–4]. However, little is known of what kind of patients would profit most from different psychotherapy modes and how different therapist techniques and interventions contribute to the patient-therapist interaction. Also, it has proved difficult to find ways to examine this in-session process. Therefore, there is a need to develop more methods to reveal what kind of treatment and what treatment techniques work best for different patients and how the various psychotherapy modes work to promote the best possible treatment effect (outcome) for each patient. Understanding the process of therapy and the impact on outcome will increase our ability to develop further our treatments, to make them more effective, and to individualize them for the needs of specific individuals [5].

Process-outcome research that examines the action of specific psychotherapies would help developing more personalized treatment. *Psychotherapy process* is primarily the actions, experiences and relatedness of patient and therapist in therapy sessions [6]. Change process might be referred to as “short-term outcome”. *Outcome* is improvements in patient-presenting problems, symptom, and functioning during therapy. Thus, process studies exploring what happens during psychotherapy sessions (e.g. single case studies, qualitative and quantitative process studies) are essential to understand the nature of psychotherapy and would be helpful when outcome is also part of the empirical investigation. On this background, there is a need for more theoretically informed quantitative assessment tools enabling researchers to explore the in-session process in psychotherapy.

In dynamic psychotherapy a theoretical assumption is that people project representational aspects of important others onto relationship with other people at present. Therefore, interpreting the patient’s relationships with other people are key components to enhance change. Those interventions might be categorized in two groups: 1) Transference interventions (TI) are when the therapist focuses on the patient’s relationship to and experience of the therapist. 2) Relational Interventions (RI) are when the therapist focuses on the patient’s relationships outside therapy. Different definitions of RI and TI are given in the literature. Some definitions of RI for example, will include TI as a variant of RI. The different definitions of these constructs are, however, beyond the scope of this paper. For our purposes, RI and TI are defined in concrete behavioral terms as an explicit interpretive reference to the patient’s ongoing relationship with other people outside therapy or the relationship with the therapist (Table 1).

**Table 1:** Five categories of Relational Interventions and Transference Interventions defined in FEST^a^

Relational interventions (RI):
1. The therapist addresses interpersonal transactions with other (s)^b^. 2. The therapist actively encourages the patient to explore thoughts and feelings about his/her relationships to other (s)^b^ including their style and behavior. 3. The therapist encourages the patient to discuss how other (s)^b^ might feel or think about the patient. 4. The therapist makes interpretive linking of dynamic elements (conflicts) in the patient’s relationships with other (s)^b^. 5. The therapist attempts to explore interpersonal repetitive patterns with important other (s)^b^ and with parental figures.
Transference Interventions (TI):
1. The therapist addresses transactions in the patient–therapist relationship. 2. The therapist encourages exploration of thoughts and feelings about the therapy and therapist’s style and behavior. 3. The therapist encourages patients to discuss how they believed the therapist might feel or think about them. 4. The therapist includes him-/herself explicitly in interpretive linking of dynamic elements (conflicts), direct manifestations of transference, and allusions to the transference. 5. The therapist was to interpret repetitive interpersonal patterns (including genetic interpretations) and link these patterns to transactions between the patient and the therapist.

*Note*. ^a^First Experimental Study of Transference-interpretations (FEST). ^b^other (s) is/are everyone except the therapist (i.e. friends, relatives, colleagues)

One example of a relational intervention might be:” You really don’t want to go to that party while your boyfriend does. Since you don’t agree with him, you feel guilt in preventing him from going. During childhood, you had to get out of your way in order not to prevent your mother reaching her goals in life. It might be that your previous feeling in relation to your mother is connected with your present feeling in relation to your boyfriend.” An extension of the RI might include an explicit reference to the patient–therapist interaction (TI) by adding:” Perhaps you accepted my suggestion of a short and time-limited therapy because you felt guilty to occupy my time here?”

In psychodynamic theory especially interpretation of the patient’s relationship to and experience of the therapist (TI), has been considered an important and frequently used specific technique [7, 8]. However, this assumption might be proved wrong as a general guideline for all patients. Transference phenomenon is also present in non-dynamic treatments, but the research in this field is sparse [9]. Patients may improve differently according to various characteristics. Some recent studies indicate that although patients on average might respond equally well in dynamic psychotherapy with or without TI, some sub groups of patients respond better when the focus is on the patient’s relations outside therapy [10, 11]. Working with the transference (Transference work; TW) more than working with relations outside therapy (Relational work; RW) is expected to trigger defense mechanisms [12, 13]. However, as far as we know, there has been little empirical exploration of the different impact for different patients on the in session process and treatment outcome of therapy with RI and TI combined versus therapy only focusing on RW.

To identify and analyze in-session effects of therapist technique and empirically establish links to outcome, we have to rely on adequate process scales [7, 14]. Different instruments for identification of therapist techniques have been developed and should be acknowledged [15]. Previously the development and use of a therapy process rating scale (Transference Work Scale; TWS) has been described and a manual was published [15, 16].

TWS was constructed to identify, categorize and explore in-session work with the transference. TWS has sub scales that rate timing, content, and valence of the transference interventions, as well as response from the patient. The inter-rater agreement on the TWS items was good to excellent. TWS might be a useful tool to explore the interaction of timing, content, and valence of transference work in predicting in-session patient response as well as treatment outcome.

### Aim

To be able to explore the in-session process in non-transference and non-dynamic therapies, the aim of the present study was, to develop a simple measure specially designed to identify the five RI categories and explore the timing, content, and valence of each intervention, as well as the response from the patient. Furthermore, the aim was to test the inter-rater reliability of the different items. Use of the scale is illustrated with two clinical vignettes.

## Method

### First experimental study of transference-interpretations

Relational Work Scale (RWS) was developed by the First Experimental Study of Transference-interpretations (FEST) research group. Clinical examples in the present paper are from therapies in FEST. The Regional Ethics Committee for health region 1 in Norway approved the study protocol and the information given to the patients. Patient material and data collected including case material were accepted for use in research and publishing as well as teaching. Written informed consent was obtained from each participant. Data are anonymized. It is not possible to reconnect the clinical material to patient identity.

The purpose of the First Experimental Study of Transference-interpretations [10, 17] was to measure the effects of Transference Work (TW) in dynamic psychotherapy. FEST was a randomized controlled trial where one hundred patients seeking psychotherapy for depression, anxiety, and personality disorders, where allocated to psychodynamic psychotherapy with low to moderate levels of TIs (the transference group; *N* = 52) or psychodynamic psychotherapy with no TIs, only RI’s (the comparison group; *N* = 48). Patients with psychosis, bipolar illness, organic mental disorder, or substance abuse were excluded. The random assignment of patients was conducted after the pre-treatment ratings were completed. Only the patients’ therapists learned the result of the random assignment procedure. Patients were assigned to one of the seven therapists, depending on availability. The clinical evaluators and therapists consisted of six psychiatrists and one clinical psychologist, all of whom had 10–25 years of experience in practicing psychodynamic psychotherapy. In the pilot phase of this study, the therapists were trained with group supervision for as long as 4 years in order to be able to administer treatment with a moderate frequency of transference interpretations (1–3 per session) as well as treatment without such interpretations with equal ease and mastery. All therapists treated patients from both groups.

The treatment was 45 min once weekly, maximum 40 sessions (1 year) [18]. In both treatment modes the therapists used RIs focusing on the patient’s relations outside of therapy. The specific techniques (i.e. categories of TI) were prescribed, for the transference group only (Table 1).

To quantify the use of the five different RI and TI categories in the two treatment groups, a global rating method using a 5-point Likert scale ranging from 0 (not at all) to 4 (very much) was used. This was chosen rather than rating the exact frequency of interventions [19, 20]. The use of the techniques differed significantly between the treatment groups. The average score of TI was 1. 7 (SD = 0.7) in the transference group, and 0.1 (SD = 0.2) in the comparison group (t = 14.8, df = 58.2, *p* < 0.0005) [10].

The outcome measures were the Psychodynamic Functioning Scales [21], Inventory of Interpersonal Problems-Circumplex version [22], Global Assessment of Functioning Scale (GAF; Diagnostic and statistical manual of mental disorders, 1987), and Symptom Checklist-90-R [23].

Based on a psychodynamic interview modified after Malan [8] and Sifneos [24], at least three clinicians rated PFS and GAF (pre-, posttreatment, and at 1- and 3-year follow-ups) and at pre-treatment also the patient’s relational functioning with Quality of Object Relations Scale (QOR) [25]. The QOR score was determined from the following three 8-point scales: evidence of at least one stable and mutual interpersonal relationship in the patient’s life, history of adult sexual relationships, and history of nonsexual adult relationships. The QOR measures the patient’s lifelong tendency to establish certain kinds of relationships with others, from mature to primitive. Inter-rater reliability for average scores of three raters was 0.84 in this study. The predetermined cutoff score for high versus low QOR was 5.00 [14].

Both treatment groups showed statistically significant change from pre-treatment to 3 year follow-up. Contrary to expectation, no significant between-group differences were revealed in FEST. Moderator analyses showed that patients with a life-long pattern of poor relational functioning measured with the QOR [17] and/or patients with personality disorders profited more in therapy with TI than without TI [10, 17, 26]. This was especially true for women, while men responded better to therapy without TI compared to women [11].

### Development of relational work scale (RWS)

Based on psychodynamic theory [13, 27, 28] and years of combined clinical practice and research experience with psychodynamic psychotherapy, the FEST-research group has developed the TWS and subsequently the RWS. Several previous adherence- and process measures have influenced the development process, and resemblance is intentional. However, the main source was the Manual for process rating from FEST [20] and the TWS [15]. While TWS was developed for identifying and categorizing TI and the interaction between therapist and patient in the Transference work, RWS was developed in order to identify and categorize RI’s and analyze the process between therapist and patient after an RI. RWS has items on the timing, content, and valence of each RI, as well as the response from the patient modelled as in the TWS. Five researchers and clinicians have participated in the theoretical discussions on constructions of items in RWS.

Two independent raters scored transcripts during the scale developmental period to determine inter-rater agreement on different items. The two raters one female rater, M.D., Ph.D. with 30 years of clinical experience and one male rater, M.D. with more than 50 years of clinical experience. Both were specialists in psychiatry. However, they were trained in different psychoanalytic/psychodynamic institutes and had dissimilar work experience. The male rater had participated in FEST from the beginning of the study while the other joined the research group after the four year study evaluations for all patients were completed.

Items concerning identification and categorization of RI were based on the five categories of RI defined in FEST (Table 1). Predefined non-transference interventions [20] and comparable process exploring items as used in TWS were included in RWS (Additional file 1).

Items assessing the degree to which RIs connect naturally to the preceding clinical material and how precise and striking the RI is [28] and items describing content of therapist interventions, such as dynamic conflict components (anxiety, defense, impulse/motives) and person components (parents, others) were included [8]. Also RWS was developed with items concerning the degree of whether the therapist was challenging or supportive, and the patient’s attempts to avoid themes, level of emotional engagement, and whether the patient showed associations or self-reflections in the response, were included [28].

Items in RWS were scored using Likert scales; 0, 1 (not at all, low degree) to 4 (high degree). Items identifying presence or absence of the five categories of RI (Item 8–12) were rated with “Yes” or “No”. The segments used for the pilot project were from previously transcribed sessions for analyses of gender differences in FEST [29]. Since the two raters had previously rated transcripts for the inter-rater analyses with TWS, and the rating method using RWS was comparable, little additional training was needed. However, first, the raters rated 10 segments from different therapies not included in the IRR analyses. During the training, ratings of one segment were discussed before next segment from another therapy was rated. No further training was conducted before rating of the segments used in the IRR-analyses.

### Ratings with RWS for inter-rater reliability analyses

Ten min segments from the middle of the mid-therapy session (session 16) were chosen to explore the RW. Transcripts from 20 of the patients in the non-transference group (10 low QOR women and 10 high QOE men), were used. Each turn of talk in the dialogue between the patient and the therapist was given consecutive index numbers in the transcript [22].

The version of RWS used for the IRR-analysis comprised 26 items (Table 2), and included two items concerning timing of the RI with the highest category score in the transcript.

**Table 2:** Rater reliability of 2 raters for the Relational Work Scale (RWS) scored on one segment from the middle of a mid-therapy session of the patients in the non-transference group (*N* = 20)^a^

Item			Rater 1 Mean (SD)	Rater 2
		Kappa	*N* (%)	*N* (%)
1	RI^b^ in transcript	1	17 (85 %)	*N* = 17 (85 %)
Identification		ICC		
		S^c^	Mean (SD)	Mean (SD)
2	Beginning IRI^d^	1	11.82 (10.67)	12.06 (10.84)
		Kappa	*N* = 17 (85 %)	*N* = 17 (85 %)
3	IRI first interaction	1	2 (10 %)	2 (10 %)
4	IRI last interaction	1	1 (5 %)	1 (5 %)
		ICC	*N* = 17 (85 %)	*N* = 17 (85 %)
		S	Mean (SD)	Mean (SD)
5	Category IRI	0.66	2.53 (1.12)	2.17 (1.29)
Timing IRI				
6	IRI connect naturally	0.33	3.00 (0.76)	2.80 (0.86)
7	IRI precise/striking	0.82	2.60 (1.12)	2.73 (1.03)
Category RI		Kappa	*N* (%)	*N* (%)
8	Category 1	0.47	3 (17.65)	7 (41.18)
9	Category 2	0.77	15 (88.24)	14 (82.35)
10	Category 3	0.85	5 (29.41)	4 (23.53)
11	Category 4	0.76	3 (17.65)	2 (11.76)
12	Category 5	1	3 (17.65)	3 (17.65)
Content				
Timing high category^e^ RI		ICC	*N* = 13 (65 %)	*N* = 13 (65 %) *N* = 17 (85 %)
		S	Mean (SD)	Mean (SD)
I^f^	Connect naturally	−0.16	3.23 (0.60)	3.08 (0.49)
II^f^	Precise/striking	0.73	2.46 (0.97)	2.54 (0.78)
		ICC	*N* = 17 (85 %)	*N* = 17 (85 %)
		S	Mean (SD)	Mean (SD)
13	T^g^ Relation other	0.89	2.35 (1.11)	2.35 (0.10)
14	P^h^ Relation other	0.96	2.82 (1.86)	2.71 (1.10)
15	T Relation parent	0.95	1.23 (1.35)	1.17 (1.33)
16	P Relation parent	0.96	0.76 (1.25)	0.76 (1.25)
17	Avoid themes	0.61	0.29 (0.69)	0.23 (0.56)
18	Symptoms T	0.63	1.35 (0.10)	1.47 (0.80)
19	Symptoms P	0.85	1.41 (0.80)	1.65 (0.79)
Valence				
20	Supportive	0.52	1.94 (0.83)	2.00 (0.87)
21	Challenging	0.66	2.11 (0.85)	1.82 (0.81)
Response				
22	Associations/self refl	0.89	2.29 (0.91)	2.35 (0.93)
23	Cooperative	0.64	2.65 (0.70)	2.47 (0.80)
24	Emotional involvement	0.62	2.47 (0.71)	2.24 (0.56)

*Note* ^a^20 patients from FEST NT group; 10 high QOR men and 10 low QOR women. 17 segments (*n* =17, 85 %) were found to contain Relational Interventions. There was complete agreement with regard to presence versus absence of Relational work in these 17 segments (Item 1 on RWS). ^b^Relational Interventions (RI). ^c^Single measure (S). ^d^Initial Relational Intervention (IRI). ^e^Timing of the first Relational Intervention with the highest category score. Deleted from final version of RWS.^f^Item deleted from the final version of RWS. ^g^Therapist focusing on (T). ^h^Patient focusing on (P)

These two items are included in the table reporting the results from the inter-rater reliability analyses and marked I (Is the timing of the RI with the highest category score in the transcript naturally connected with the preceding clinical material?) and II (How and precise and striking is the therapist’s RI?) in Table 2.

### Statistical analyses

The IRR was estimated with the coefficient Kappa [30] for category scores and with the Intra Class Correlation (ICC) [31] (two-way random consistency) for ordinal scores. According to Landis and Koch kappa values greater than 0.75 are interpreted as excellent agreement, 0.60–0.75 as good, between 0.40 and 0.60 as fair and below 0.40 indicates poor agreement [30]. The statistical analyses were done using SPSS version 18 SPSS Inc., 2011).

## Results

The RWS showed good inter-rater reliability for almost every item. The raw *N* and frequencies for occurrence of each of the categorical variables are listed in Table 2. The raters identified RI in 17 of the 20 segments (kappa = 1). Except for Item 6 (whether the therapist’s initial RI was connected naturally to the preceding clinical material, content and time line), and the Item marked “I” in Table 2 (whether therapist’s RI with the highest category score in the transcript connect naturally to the preceding clinical material), the scoring agreement was good to excellent. On item 6 the IRR was low. On Item I the IRR was negative, however, the two raters scored nearly identical.

On most items, the ratings were normally distributed for both raters. Ratings on the items concerning whether the therapist (Item 13) or patient (Item 14) refer to the patient’s relationship to parents, and whether the therapist points out that the patient attempts to avoid themes (Item 17) the percent of 0-scores was high for both raters; 41.2 and 47.1 % on Item 13 for rater 1 and 2 respectively, 64.7 and 82.4 % on Item 14 and 17 respectively for both raters.

Because of the negative IRR on one of the items concerning timing of the RI with the highest category score in the transcript, both Item I and Item II (Table 2) (Is the timing of the RI with the highest category score in the transcript naturally connected with the preceding material (I) and precise and striking (II) (Table 2) were deleted in the final version of RWS (Additional file 1).

During the rating process, examples of the five different categories of RIs were identified and are provided in Table 3.

**Table 3:** Examples of Relational Interventions (RI)^a^

RI Category 1: The therapist addresses interpersonal transactions with others without any linking to analogous transactions in session.	
Therapist	However you will keep up appearances. Pretending that everything is okay with you when you meet him.
RI Category 2: The therapist actively encourages the patient to explore thoughts and feelings about his/her relationships to other (s) including their style and behavior.	
Patient	Our children prefer being with my wife. It often seems fine and lively when they are together, while it often becomes awkward when they are with me.
Therapist	How do you feel about that experience?
Patient	It is sad when I think about these situations and what I lose.
RI Category 3: The therapist encourages the patient to discuss how other (s) might feel or think about the patient.	
Patient	When I unexpectedly come home to visit a Sunday, my mother would say: “it’s been a long time not seeing you”.
Therapist	Could it be an indication that she is happy? That she misses you more than that she blames you?
Patient	Yes, I think so. She actually is quite supportive. However, I don’t really need to meet my parents as often now. It is more like I meet them when I want to. But if my relationship to my girlfriend ends, I think I would visit my parents. Then I will need some kind of comfort, as I always get there.
RI Category 4: The therapist makes interpretive linking of dynamic elements (conflicts) in the patient’s relationships with other (s)	
Patient	He says he is very fond of me and would really like to have a relationship with me.
Therapist	So you are afraid of being let down, that he would lose interest in you when he gets to know you better?
Patient	Mmm mmm (confirms)
Therapist	This fear that people who matter to you would not bother about you. Did you always have this fear or did it come gradually?
Patient	I think I have had this feeling for a long time, as long as I can remember, but I have never really thought about it before.
RI Category 5: The therapist attempts to explore interpersonal repetitive patterns with important other (s) and with parental figures.	
Therapist	You say that your boss, in this case, is breaking the regulations. Still you do not confront him, rather you feel like it’s your fault; that you have been leading him to it. You defend your boss and suppress your protest. Could it be helpful for you to see this as sort of an extension of the relationship with your father? When facing your father you did not share your opinions or views, that is, if you assumed that your father did not agree. You were afraid of being rejected and losing your father’s strong support and positive recognition.
Patient	Yes. It is good to bring up my anger against my boss in this matter, because I put the blame on my anger easily and then I experience that he could not have acted in any other way and that it is almost my fault. It is of course not logical, but that is how I feel. Yes, it is a bit like with my father, I never really expressed my disagreement or criticism towards him. So what I feel now is discomfort, like a knot in my stomach, I want to put the whole case aside.

^a^All clinical examples are identified in the First Experimental Study of Transference-interpretations (FEST). Written informed consent was obtained from each participant

### Two vignettes illustrating the use of the relational work scale

#### Case 1 (Low QOR woman treated with TI)

Annie Lowland (not her real name) worked as a headmaster. She was 52 years, lived in a stable marriage with four grown up children. Annie Lowland grew up with three younger sisters. She was from the northern part of Norway. Her father was stable, but distant. Her mother was perceived as unpredictable and mean. In three periods during her first four years, Annie lived with relatives because her mother was “tired”. When Annie was a grown up, her mother was treated for periodic alcohol abuse.

She emphasized that she wanted to raise and educate her children in another way than she was brought up herself. Her first child died, from meningo-encephalitis, as a toddler. Through recurrent episodes of depression, she accused herself for the boy’s death. Three years ago she was successfully treated for breast cancer with breast conserving surgery. However, after the operation, she felt she had failed at work, as a mother, wife and woman. She felt insecure, with low self-esteem. She was depressed, felt her quality of life was low and hoped therapy would help.

Reflecting her interpersonal problems, Annie Lowland was rated with low QOR.

#### Course of treatment

Annie Lowland was randomized to dynamic psychotherapy without TI. She attended 39 sessions. The focus for the therapy was expected to be her relationship with her mother and her low self-esteem.

For a short example from the dialog late in therapy, please see Table 4. Table 4 illustrates how the IRR-raters would evaluate the presence and timing of RIs in this segment, as well as the themes in the dialogue and the patient’s response.

**Table 4:** Segments with Relational Interventions (RI) from the patient–therapist dialogue in a session from late in therapy rated with Relational Work Scale

Individual speaking	Parts of dialogue	(RI): Category 1–5
		Rated 0–4:
		(T): Timing
		(C): Content
		(V): Valence
		(R): Response
Therapist	Your relationships with your colleagues seem to have changed.	RI:2
		C: Other (3)
		V: Supportive (0),
		Challenging (2)
Patient:	In my job, control and management is really important. However, I have a feeling that I exaggerate the control-part of my work. I don’t just control and organize the school; I control the teachers in detail too. I don’t like this side of myself.	C: Other (1)
		R: Associations/self reflections (3),
		Cooperative (3),
		Emotional (4)
Therapist	Maybe you feel that you’re not any longer a colleague, but only a manager and boss. You might see similarities between yourself and the way your mother acted. She was controlling and interfered in others business. I wonder what impact the way you have experienced your mother has had on your self-image.	RI: 5
		T: Connect natural (3) precise/striking (3)
		C: Other (2)
		Parents (2)
		V: Supportive (1), Challenging (4)
Patient:	It gives me a feeling of not being good enough. After the cancer operation I doubt my value as a woman even though I know it’s irrational. However, when receiving positive response at work, I get a good feeling of being kind, clever and pretty.	C: Other (2), Symptoms (4)
		R: Associations/self reflections (1),
		Cooperative (1), Emotional (2)

Table 4 shows only a very short excerpt from a session and ratings of the segment are solely meant for illustrating the use of RWS. The two raters identified RIs of category 2 (first RI in transcript) and 5 (second RI). Timing is only rated for the second RI because the first RI is the first patient-therapist interaction in the transcript (Item 3). The first RI was delivered in a less supportive and challenging way rated 0 and 2 (average 1) and 2 and 4 (average 3) respectively by the two raters. The RI in category 5 might have been experienced as quite demanding by Annie Lowland. The therapist asked Annie to explore her interpersonal repetitive patterns towards her colleagues and also compared this pattern with how Annie experienced her mother’s negative influence on Annie’s self-image. Thus, the therapist focused on other (average 2.5) and mother (2). The patient responded with associations/self-reflections, however, more freely after the first than the second RI (rated 3 and 1 respectively). She also seemed to be more cooperative and emotional involved after the first (3 vs. 4) than the second (1vs. 2) RI. After the challenging category 5 intervention the patient’s response was to a little degree focused on other (2) and Annie did not follow the therapist’s intervention by exploring her relationship with her mother. After the second intervention, the patient seemed to be more distanced. She responded with talking more about others, was less emotional involved, focused more on her own symptoms, and was rated less cooperative.

After treatment, Annie Lowland vaguely described that she experienced improvement and fewer symptoms. She still struggled with her collegial relations at work. However, she described that her self-esteem in relation to her husband had improved a little.

#### Case 2 (High QOR man treated without TI)

Randolph Highland (not his real name) was a 43 year old geologist working in the petrol industry in Norway.

He married young and had two children 10 and 14 years old. Over the years, he felt more and more rejected by his wife. She was verbally strong and he responded by withdrawing himself from discussions. He had two good mutual friendships and was highly valued as a colleague because he was always helpful and took on more than his share of the work load.

Randolph Highland grew up as the youngest of four on a farm in the eastern part of Norway. The oldest sibling was a sister proud to be the one inheriting the farm. He also had two older brothers. However, he seldom was welcomed to participate in their activities. His mother was a dominating woman who punished the family members with rejection if they were not submissive to her. His father did not oppose to Randolph’s mother. Randolph Highland developed a behavior characterized by low assertiveness and a tendency to avoid conflicts.

He felt that his quality of life was low. However, he expected therapy would be helpful. He was rated with high QOR.

#### Course of treatment

Randolph Highland was also randomized to dynamic psychotherapy without TI. He came to 36 sessions. Based on the background information it was expected that the therapist would focus on his inhibited aggression, fear of rejection and guilt if he insisted on his own opinion.

A short segment from mid-treatment is shown in Table 5. Table 5 illustrates how the IRR-raters would evaluate the presence and timing of RIs, the themes in the dialogue and the patient’s response.

**Table 5:** Segments with Relational Interventions from the patient–therapist dialogue in a mid-therapy session rated with Relational Work Scale

Individual speaking	Parts of dialogue	(RI): Category 1–5
		Rated 0–4:
		(T): Timing
		(C): Content
		(V): Valence
		(R): Response
Therapist	You have previously focused on whether you wanted to continue to work with your colleagues in that committee.	RI: 1
		C: Other (4)
		V: Supportive (1), Challenging (1)
Patient	I planned to refuse reelection to the committee. However, I was persuaded to continue by the other members. I kind of regret accepting the position because it takes time from other things I want to do like participate in the literature group with my friends.	C: Other (3)
		R: Associations/self-reflections (3), Cooperative (3), Emotional (3)
Therapist	Then the other members in the committee think you are a great chap. You care for your family, colleagues and friends, but do you care about your own needs?	RI: 2 and 3
		T: Connect natural (3)
		Precise/striking (4)
		C: Other (4)
		V: Supportive (3)
		Challenging (1)
Patient	At home my kids know my wife’s opinion, but not mine. They want me to be more explicit. On the other side, I get guilty conscience and fear of them getting angry if I am too opinionated.	C: Other (3)
		R: Associations/self reflections (2), Cooperative (3), Emotional (2)
Therapist	Yes, they have even told you to stop care so much about them and start prioritize what is necessary for yourself, for example to buy yourself some new clothes. Do you think that is a common concern among children and teenagers nowadays?	RI:2
		T: Connect natural (2)
		Precise/striking (3)
		C: Others (3)
		V: Supportive (1), Challenging (3)

The raters of the segment from Randolph Highland’s session identified RIs in category 1, 2 and 3. The timing of the first RI is not decided since this is the first patient-therapist interaction in the transcript. The timing of the second and third RI is on average 3. The first RI was a preparing intervention where the therapist addressed previous interpersonal transactions with other people. In the first RI the therapist was a little supportive as well as a little challenging (1), in the second RI the therapist was more supportive (3) and still a little challenging (1). In the third RI the therapist was less supportive (1) and more challenging (3). This is the last patient-therapist intervention. Therefore we don’t know how the patient responded to this intervention in the transcript. In the second RI the therapist actively encouraged Randolph Highland to explore thoughts and feelings about his relationships to others (colleagues at work; rated 4) and how he imagined they might feel or think about him (RI category 2 and 3). The timing of the intervention was good (3.5). Randolph Highland responded with associations and self-reflection (rated on average 3), was cooperative (3) and emotionally involved (2.5). Neither the therapist, nor the patient mentioned the patient’s symptoms directly. They both focused on Randolph Highland’s relationships with other people.

After therapy Mr. Highland described that he had made huge changes in his work load and his relations at work. He still participated as a good team worker, but did not any longer compensate when colleagues did not complete their duties. He was intellectually aware of his still somehow submissive relational pattern with his wife, and articulated that he was satisfied and that he did not want to change this further.

There are obvious limitations in drawing any conclusions from ratings with RWS of these short segments from the two vignettes. However, the available material rated with RWS indicates different processes and therapist –patient interactions in the two therapies. Annie Lowland, who at pre-treatment showed difficult interpersonal relationships, experienced more challenging and less supportive therapist interventions. In the segment from Randolph Highland’s therapy both the therapist and the patient talked about Randolph’s relations with other people, however, not about his relationships with his parents. When Annie’s therapist pointed at the patient’s relationship with her mother and compared elements in the mother’s and Annie’s, relational patterns, Annie refused to explore this, while she began talking about her symptoms. These ratings might be hints that point towards a possible understanding of why Annie, while being challenged by her therapist, preferred talking about symptoms rather than relationships and did not experience improved relations with other people. Randolph might have experienced the therapist interventions more supportive and less challenging, and managed to explore interpersonal relationships to a greater extent. This may have resulted in improved relations with other people. No exploration of his image of his dominating mother was present in this short segment. However, if this interaction was representative for the therapy, the lack of focus on the repetitive interpersonal pattern might have been a hindrance for him in further resolving his relationship with his dominating wife.

## Discussion

The Relational Work Scale was specifically developed to identify and explore in more detail Relational Interventions and the in-session process after such interventions and thus, explore the relational work. Five categories of RIs were used in RWS, to delineate and operationalize the out of therapy relational constructs. Items on timing of therapist interventions, content, and the interaction between patient and therapist were used in the scale which has comparable items as the previously published Transference Work Scale.

A limitation is that RWS was developed within the FEST research group on items defined by interventions prescribed in the treatment manual [20]. The manual was also developed by the same research group. One of the researchers had participated in the study also as a rater and therapist and knew the study and the interventions very well. In the present study, the adherence to the manual was high. Another limitation might be that the ratings with RWS are performed on 10-min segments. To know whether RWS might be a useful tool for other research groups, IRR of RWS needs to be tested on transcripts from other therapies in other studies also with longer transcripts. As with TWS [16], RWS was promising with regard to achievement of inter-rater agreement. Due to limited resources, however, the number of rated sessions in the inter-rater analyses of RWS was limited.

Even though the two raters differed in psychodynamic training, theoretical background, and work experience the rater agreement for nearly all the RWS items in the present study was good to high. RWS is a focused and short process measure and is seemingly not too time consuming. Together with TWS, RWS may be used for creating datasets from a larger number of sessions or segments of sessions. The two vignettes also indicate that through rating of transcribed segments or whole sessions, use of differing therapist interventions and patient responses might be revealed. Different categories of RI and other features/aspects of relational work may relate differently to outcome. Previously reported interaction effects of RI/TI and sub-groups of patients need to be further explored with process-outcome studies. A focus on the unexpected positive effect of RI for some patient groups could be further investigated [11, 26, 32]. RWS might prove especially suitable for intensive case studies combining quantitative and narrative data.

## Conclusion

The Relational Work Scale was specifically developed to identify and explore in more detail Relational Interventions and the in-session process after such interventions. The inter-rater agreement was good to high. RWS is a focused and short process measure. This study suggests that RWS might be a potentially useful tool to identify RI as well as explore the interaction of timing, category, and valence of relational work in psychotherapies that focus on the patient’s relations with other people than the therapist.

## Additional file

Additional file 1: Relational Work Scale (RWS). (DOCX 16 kb)

## Abbreviation

FEST: First experimental study of transference–interpretations
IRR: Inter rater reliability
QOR: Quality of object relation score
RI: Relational interventions
RW: Relational work
RWS: Relational work scale
TI: Transference intervention
TW: Transference work
TWS: Transference work scale
