# Supplementary material for: Relational interventions in psychotherapy: development of a therapy process rating scale
Source: BMC Psychiatry. 2016 Sep 6;16(1):310. doi: 10.1186/s12888-016-1021-4 (PMC5011870; doi:10.1186/s12888-016-1021-4)
Supplement: Additional file 1: — Relational Work Scale (RWS). (DOCX 16 kb) [file 12888_2016_1021_MOESM1_ESM.docx]

**Additional file 1**

**Relational Work Scale (RWS)**

**Identification**

1. Are there any relational interventions^1^ in the transcript from

patient________,session________and segment________? YES NO

*If YES, answer the following questions:*

2. What is the index number at the beginning of the Initial Relational Intervention (IRI)?________

3. IRI is the first therapist-patient interaction in the transcript. YES NO

4. IRI is the last therapist-patient interaction in the transcript YES NO

5. What is the category of the IRI? _________

**Timing of the Initial Relational Intervention (IRI)**

6. To what degree does the therapist’s IRI connect naturally to the preceding clinical material, such as content and time

line, allusions to the relational aspects and other relevant issues? 0 1 2 3 4^2^

7. How precise and striking is the therapist’s IRI? 0 1 2 3 4

**Category of the Relational Interventions (RI) in the Relational Work (RW)**

8. Does the RW include RI of category 1? YES NO

9. Does the RW include RI of category 2? YES NO

10.Does the RW include RI of category 3? YES NO

11.Does the RW include RI of category 4? YES NO

12.Does the RW include RI of category 5? YES NO

**Content in the Relational Work^3^ (RW)**

13. To what degree does the therapist refer to the patient’s relation to others? 0 1 2 3 4

14. To what degree does the patient refer to the patient’s relation to others? 0 1 2 3 4

15. To what degree does the therapist refer

to the patient’s relation to parental figures? 0 1 2 3 4

16. To what degree does the patient refer to the

patient’s relation to parental figures? 0 1 2 3 4

17. To what degree does the therapist point out

the patient’s attempt to avoid themes in the session

in order to control unpleasant emotions and thoughts? 0 1 2 3 4

18. To what degree does the therapist refer to the patient’s symptoms? 0 1 2 3 4

19. To what degree does the patient refer to the patient’s symptoms? 0 1 2 3 4

**Valence – in the Relational Work (RW)**

20. To what degree does the therapist make use of supportive interventions? 0 1 2 3 4

21. To what degree is the therapist challenging in the interventions? 0 1 2 3 4

**Response – in the Relational Work (RW)**

22. To what degree does the patient

express associations and/or self-reflections in the RW? 0 1 2 3 4

23. To what degree does the patient show active cooperative engagement? 0 1 2 3 4

24. Identify with the patient:

What is the highest level of emotional involvement? 0 1 2 3 4

^1^*Relational Intervention (RI):*The therapist addressed transactions in the patient’s relationships with others.

^2^0 (not at all), 1 (low degree), 4 (high degree).

^3^*Relational Work (RW)* is the interaction between the therapist and the patient following the initial RI and continues throughout the transcript.
